# Investigation of the splitting tensile performance of Rock-Filled Concrete based on mesoscopic modeling: Effects of the interfacial transition zone and rockfill characteristics

**DOI:** 10.1371/journal.pone.0351188

**Published:** 2026-07-22

**Authors:** Qian Li, Ning Liu, XiaoLing Tang, Peng Chen, YouBin Li, Tao Yang

**Affiliations:** College of Civil Engineering, Guizhou University, Guiyang, Guizhou, China; China Construction Fourth Engineering Division Corp. Ltd, CHINA

## Abstract

Rock-filled concrete (RFC) is a three-phase heterogeneous composite composed of large-size rockfill, self-compacting concrete (SCC), and the interfacial transition zone (ITZ) between them. Its mechanical performance is jointly affected by the rockfill skeleton effect and the properties of the ITZ. Existing studies still provide insufficient understanding of how interfacial properties and rockfill characteristics influence the splitting tensile performance and damage evolution mechanism of RFC. In this study, splitting tensile tests of RFC were conducted based on an actual engineering project, and an engineering-scale three-dimensional mesoscopic finite element model was established based on the experimental results. The effects of ITZ strength, rockfill strength, and rockfill particle size on the splitting tensile mechanical response, damage evolution process, and failure morphology of RFC were investigated. The results show that when σ_ITZ_ ∕ σ_scc_ increased from 0.2 to 1.0, the splitting tensile strength of RFC increased by 36.75%; when σ_Rock_ ∕ σ_scc_ increased from 1.0 to 5.0, the splitting tensile strength increased by 39.65%. The splitting tensile strength of RFC increased with increasing ITZ strength and rockfill strength and gradually approached saturation, with threshold values of σ_ITZ_ ∕ σ_scc_ = 0.8 and σ_Rock_ ∕ σ_scc_ = 4.0, respectively. The influence of rockfill particle size on splitting tensile strength showed a trend of first increasing and then decreasing. Compared with a single particle size distribution, a graded particle size distribution improved the rockfill skeleton structure and more effectively enhanced the splitting tensile performance of RFC. Overall, improving ITZ quality and adopting a properly graded rockfill particle size distribution are important approaches for enhancing the splitting tensile performance of RFC, and the findings provide a theoretical basis for RFC material design and engineering applications.

## 1. Introduction

Rock-filled concrete (RFC) is a three-phase heterogeneous composite composed of rockfill, self-compacting concrete (SCC), and the interfacial transition zone (ITZ) between them [1,2]. Its construction process generally involves placing rockfill into pre-erected formwork to form a skeleton structure, followed by the casting of SCC, which flows under its own weight to fill the voids among the rockfill and bond the different phases into an integrated composite system [3]. This construction method is similar to that of two-stage concrete and preplaced aggregate concrete, as all involve the prior formation of an aggregate or rockfill skeleton, followed by the filling of voids with grout or cement-based materials [4,5]. In addition, pervious concrete (PC) also exhibits a distinct aggregate skeleton structure; however, it is typically produced with a low sand content or without fine aggregates, thereby retaining a large number of interconnected pores between aggregate particles. Its pore structure and skeleton formation mechanism therefore differ from those of RFC. Compared with conventional concrete, RFC requires no vibration during construction and has a relatively simple construction process, thereby reducing energy consumption and construction disturbance. Moreover, the use of rockfill partially replaces cementitious materials, which helps reduce material cost and carbon emissions and provides economic and environmental advantages [6]. Owing to these advantages, RFC has been widely used in dam engineering [7].

The mechanical performance and failure mechanism of RFC are critical to the safety assessment and service reliability of engineering structures. Tensile strength is one of the key parameters in concrete structural design and crack control [8], and the imbalance between tensile capacity and restrained stress directly leads to concrete cracking. In engineering applications, RFC forms a rockfill skeleton using large-size rocks, typically ranging from 300 to 1000 mm, with a rockfill ratio of up to 55% [3]. This results in extensive contact interfaces between the rockfill and SCC, causing the internal load transfer, stress redistribution, and crack propagation behavior of RFC to differ from those of ordinary concrete [9,10].

Previous studies have shown that aggregate characteristics can influence the damage evolution and macroscopic mechanical properties of concrete by regulating the microstructural compactness and interfacial bonding performance of the ITZ. Owing to differences in surface roughness, mineral composition, elastic modulus, and chemical activity, different types of aggregates can alter the mechanical interlocking, chemical bonding, and local stress concentration state at the aggregate–paste interface, thereby affecting the initiation and propagation of microcracks in the ITZ region [11]. Differences in aggregate particle size distribution and surface morphology can further modify the spatial distribution of the ITZ, the extent of the fracture process zone, and the crack deflection path [12–14]. For recycled aggregate concrete, the presence of old mortar layers and multiple weak ITZ weakens the interfacial bonding performance, promotes interfacial crack propagation, and causes the damage process to exhibit more pronounced brittle characteristics [15]. Meanwhile, supplementary cementitious materials (SCMs), such as fly ash and silica fume, as well as nano-modified materials such as nano-SiO_2_, can improve the compactness of the matrix and interfacial regions through pozzolanic reactions, micro-filling effects, and nano-reinforcement effects, thereby reducing initial microcracks and enhancing the mechanical properties and crack resistance of concrete [16].

In addition to the indirect regulation of the ITZ by aggregate characteristics and SCMs, the intrinsic parameters of the ITZ and the inherent characteristics of aggregates can also directly affect the mechanical response and failure behavior of concrete [17–20]. K. M. Lee et al. [21] showed that an increase in ITZ volume fraction or a decrease in ITZ elastic modulus weakens the overall elastic modulus of concrete, and that the elastic modulus of concrete is more sensitive to variations in ITZ volume fraction. Zhou et al. [22] further indicated that ITZ strength significantly affects the dynamic tensile failure mode and mechanical properties of concrete; increasing ITZ strength can reduce the number of cracks and improve the splitting tensile strength and overall stiffness of concrete. At the same time, as a major component of concrete, aggregates themselves have an important influence on concrete performance [23–25]. Studies by Turan Özturan et al. [26] and K. P. Vishalakshi et al. [27] showed that coarse aggregate type has a significant influence on the compressive and tensile strengths of high-strength concrete, whereas the mechanical properties of low-strength concrete are mainly controlled by the ITZ and mortar matrix. Based on mesoscale simulations, Liu et al. [28] further reported that aggregate content and maximum aggregate size affect the dynamic splitting tensile strength and size effect of concrete. At low strain rates, the splitting tensile strength decreases with increasing specimen size, while increasing aggregate content and particle size can increase the nominal strength of concrete.

The above studies provide an important basis for understanding the effects of aggregates, SCMs, and the ITZ on the mechanical properties and failure behavior of ordinary concrete. However, owing to the unique large-size rockfill skeleton structure, high rockfill volume fraction, and extensive rockfill–SCC interfaces in RFC, its heterogeneity, skeleton effect, and interfacial damage characteristics are more pronounced than those of ordinary concrete. Therefore, the findings obtained from studies on aggregate characteristics and ITZ behavior in ordinary concrete cannot be directly applied to RFC. Accordingly, investigating the effects of ITZ strength and rockfill characteristics on the splitting tensile strength and damage failure behavior of RFC is of practical engineering significance.

RFC is a mass concrete material with pronounced heterogeneity in its internal structure. The use of engineering-scale specimens in studies of its mechanical behavior can more accurately characterize the actual stress state and failure characteristics of the material. However, large-scale experiments require substantial investment in specimen preparation, loading conditions, and the number of repeated tests, and they are also time-consuming. These factors impose certain limitations on the systematic analysis of key parameters such as the ITZ and rockfill particles. Meanwhile, mesoscale numerical simulation can effectively capture the failure process of RFC by explicitly characterizing its three-phase constituents, thereby providing an efficient and controllable approach for establishing the correlation between macroscopic mechanical behavior and mesoscale failure mechanisms.

Therefore, a cubic finite element model of RFC with a side length of 2000 mm was constructed using a three-dimensional spatial reconstruction method.The effects of ITZ strength and rockfill properties on the mechanical response and damage mechanism of rock-filled concrete under splitting tensile loading were quantitatively analyzed. Numerical splitting tensile tests of RFC were then conducted by assigning different values of σ_ITZ_/σ_SCC_ and σ_Rock_/σ_SCC_,as well as varying rockfill parameters.The mechanical response, failure morphology, and damage evolution characteristics of RFC under splitting tensile action were revealed, providing a mechanical basis for optimizing the tensile performance of RFC, selecting material parameters, and supporting engineering applications.

## 2. Experimental program

### 2.1 Specimen preparation and test method

**Fig 1:**
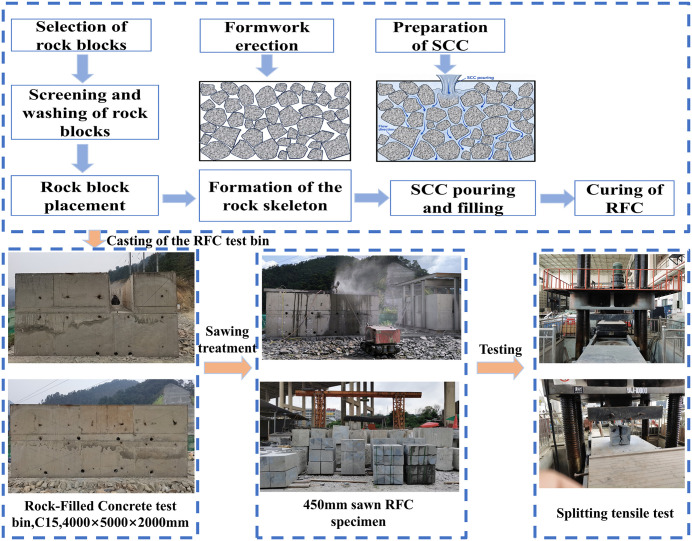
Workflow for the preparation and splitting tensile testing of RFC specimens.

This experimental study was conducted based on a reservoir dam project in Guizhou Province, China. The RFC test bin was cast in situ using the same construction method and raw materials as those used in the project. The test bin had dimensions of 5000 mm × 4000 mm × 2000 mm, with a rockfill ratio of 50%. After curing for 90 d, the test bin was cut into cubic specimens with a side length of 450 mm, which were then subjected to splitting tensile tests using a 10000 kN computer-controlled electro-hydraulic servo compression testing machine. Bearing strips with dimensions of 15 mm × 15 mm × 500 mm were used during loading. The preparation of the RFC test bin, specimen cutting, and splitting tensile testing process are shown in Fig 1. Field site access and specimen preparation were approved by QIANDONGNAN WATER INVESTMENT GROUP CO., LTD.

The main materials used for the test bin included P.O 42.5 ordinary Portland cement, Grade II fly ash, manufactured sand, gravel, admixture, and rockfill. The average compressive strength of the rockfill was 83.77 MPa, and the strength grade of the self-compacting concrete was C15. The mix proportion of SCC is listed in Table 1. Unlike ordinary concrete, which is typically cast as an integrated material after uniform mixing, RFC is prepared by first placing large-size rockfill to form a skeleton, followed by casting SCC to fill the voids among the rockfill and bond the skeleton into an integral composite. Since the rockfill skeleton occupies approximately 50% of the material volume, the volume fraction of cement-based materials in RFC is significantly lower than that in ordinary concrete. The cement consumption per unit volume of ordinary concrete and RFC was therefore compared, as shown in Table 2.

**Table 1:** SCC mixing ration (kg/m^3^).

Material	Cement	Ash	Stones	Sand	Water	Admixtures
C_90_15	135	325	753	883	175	5.9

**Table 2:** Comparison of cement consumption between ordinary concrete and RFC.

Concrete type	Material composition	Rockfill volume fraction	Cement-based material volume fraction	Cement consumption	Reduction relative to ordinary concrete
RFC	Rockfill skeleton filled and bonded by SCC	50%	50%	67.5 kg/m^3^	81.09%
Ordinary concrete	Cement-based matrix with fine and coarse Rockfillregates uniformly mixed	0	100%	357.0 kg/m^3^	**–**

Note: The cement consumption of ordinary concrete was taken from the experimental mix proportion of C30 ordinary concrete reported in Ref. [29]. In the RFC used in this study, the strength grade of SCC was C15; however, after SCC and rockfill were combined to form RFC, the compressive strength of the specimens was approximately 30 MPa. Therefore, C30 ordinary concrete was selected as the reference for comparison.

### 2.2 Experimental results

#### 2.2.1 Failure mode.

**Fig 2:**
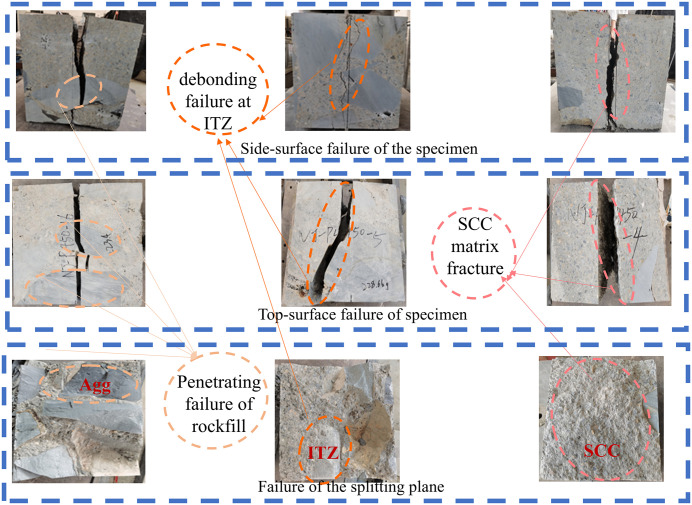
Typical splitting tensile failure mode of RFC.

Fig 2 shows the failure conditions of the top surface, side surface, and splitting plane of the RFC specimens after the splitting tensile tests. It can be observed that, under splitting tensile loading, the RFC specimens developed a through crack along the concentrated loading line, and their failure morphology was generally consistent with that of conventional concrete [30].

According to the distribution of rockfill in different specimens, when no rock particles were present near the concentrated loading line, tensile stress concentration developed in the central region of the specimen. Cracks first initiated in the SCC matrix near the loading axis and then propagated toward the upper and lower ends. In this case, the failure was mainly characterized by fracture of the SCC matrix, and the crack path was relatively straight, indicating that the SCC matrix governed the crack initiation and propagation process.When rock particles were distributed near the concentrated loading line, the main crack path changed from relatively straight to tortuous and tended to propagate along the rockfill contour. This was mainly because the crack-arresting effect and stress redistribution induced by the rockfill altered the local crack propagation direction. As the relatively weak region between the rockfill and the SCC matrix, the ITZ was prone to local stress concentration due to its relatively weak interfacial bonding performance and the stiffness mismatch between the two materials. Consequently, cracks tended to initiate preferentially in the ITZ region and propagate along the interface, eventually leading to interfacial bond failure. As the load continued to increase, when the local stress concentration exceeded the bearing capacity of the rockfill, the crack further penetrated into the rockfill and propagated through it, ultimately forming rockfill penetration failure. Meanwhile, observations of the splitting plane revealed that the internal failure of the specimens was complex. This complexity was mainly attributed to the random distribution of rock particles and their mutual interaction through interlocking and occlusion. Under external loading, rock particles at different locations exhibited distinct deformation and failure characteristics, thereby increasing the complexity of both the splitting-plane failure and the mechanical response.

These observations indicate that the splitting tensile failure of RFC results from crack initiation, propagation, and penetration within the three-phase heterogeneous structure composed of the SCC matrix, ITZ, and rockfill. The ITZ, as a relatively weak region, plays an important role in crack initiation and early propagation. The rockfill alters the crack propagation path through crack arrest, crack deflection, and local load-bearing effects, and enhances the local heterogeneity of damage evolution, ultimately causing RFC specimens to exhibit a more complex failure morphology than ordinary concrete.

#### 2.2.2 Splitting tensile strength.

**Fig 3:**
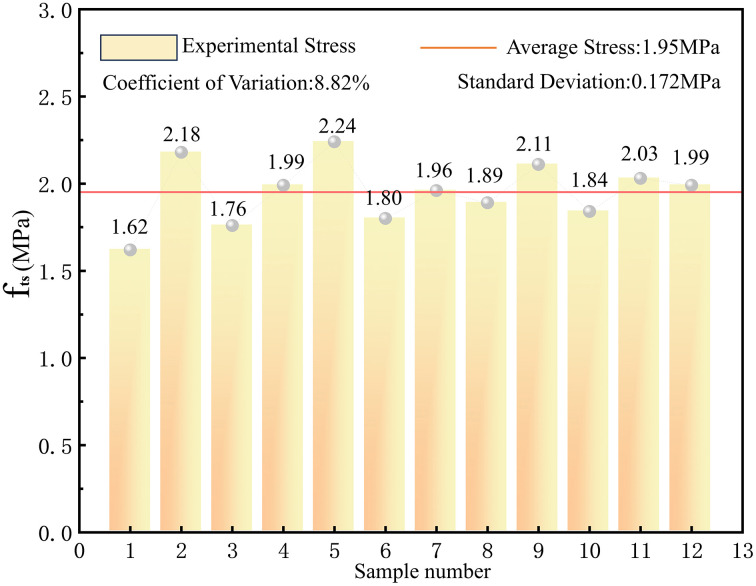
Splitting tensile strength of 12 groups of specimens.

The splitting tensile strength results of the cut specimens are shown in Fig 3. The average splitting tensile strength of the 12 RFC specimens was 1.951 MPa, with a standard deviation of 0.172 MPa and a coefficient of variation of 8.82%, indicating a certain degree of scatter among different specimens. Compared with the splitting tensile strength of ordinary concrete at the same compressive strength level, the average splitting tensile strength of the RFC specimens was approximately 63.6% of that value, corresponding to a reduction of about 36.4% [29]. Combined with the failure morphology discussed above, this difference is mainly related to variations in the internal rockfill distribution and weak interfacial regions within the specimens. Since RFC contains a skeleton formed by large-size rockfill and extensive rockfill-SCC interfaces, its damage and failure under loading are jointly affected by rockfill crack-arresting effects, interfacial debonding, and local stress redistribution. As a result, the splitting tensile strength of RFC exhibits relatively large scatter, and its splitting tensile behavior differs from that of ordinary concrete.To minimize the influence of uncontrollable factors and ensure the reliability of the test results. the average tensile strength of the 12 specimens was taken as the representative value,ƒ_ts._

## 3. Three-Dimensional mesoscopic model of RFC

### 3.1 Establishment of the geometric model

**Fig 4:**
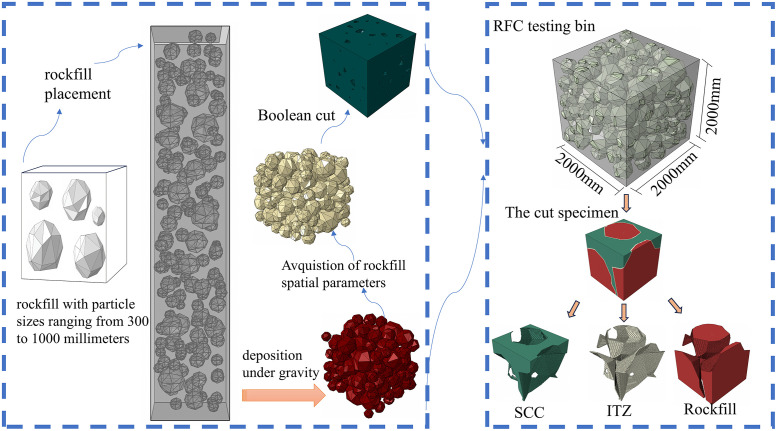
Development procedure of the finite element model for RFC.

Compared with conventional concrete, rock-filled concrete is characterized by a high rockfill ratio and large aggregate size. Conventional modeling methods have difficulty achieving a rockfill ratio of approximately 55%. The three-dimensional spatial reconstruction method proposed by Liang et al. [31]can generate mesoscopic RFC models with high rockfill ratios. In this study, this method was adopted to establish a three-dimensional mesoscopic finite element model of rock-filled concrete with material parameters consistent with those used in the actual engineering project. The model dimensions were 2000 mm × 2000 mm × 2000 mm, the rockfill ratio was 50%, and the rockfill size ranged from 300 to 1000 mm. The specific modeling procedure was as follows(Fig 4):

First, based on the three-dimensional random polyhedral modeling method proposed by Zhang et al. [32], irregular convex polyhedral aggregates with particle sizes of 300 - 1000 mm were generated. A lattice of points was then created in a gravity-deposition box measuring 2000 mm × 2000 mm × 1000 mm for aggregate placement.

Second, gravitational loading was applied to the aggregates, allowing them to fall freely and form a rock skeleton with a rockfill ratio of 50%. The displacement and rotation angle of each rock particle in the rock skeleton were extracted using a Python script to establish the ITZ.

Finally, the SCC phase filling the voids among the aggregates was obtained using a Boolean cutting method, resulting in a cubic RFC test bin with a side length of 2000 mm. After virtual cutting of the test bin, specimens measuring 450 mm × 450 mm × 450 mm were obtained through mesh mapping for the numerical simulation tests. All elements used in this study were hexahedral elements (C3D8R).

**Fig 5:**
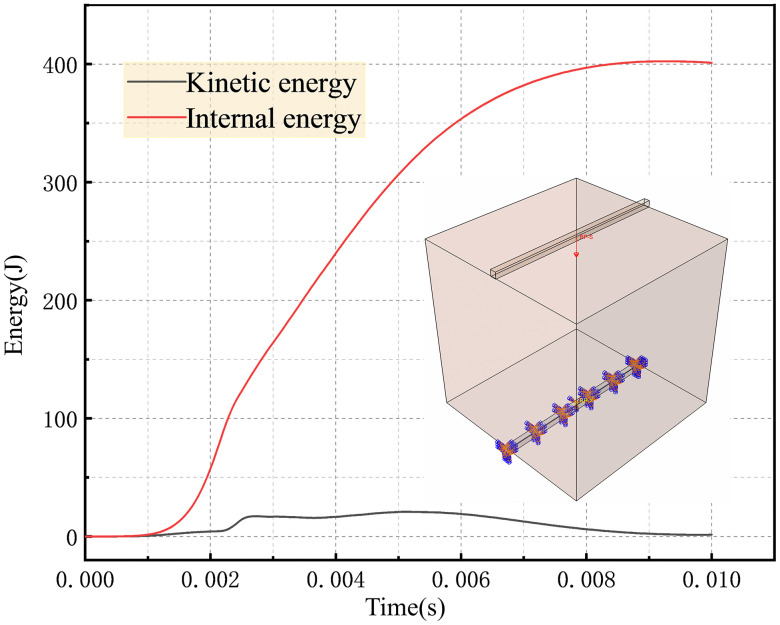
Kinetic and internal energy time histories; the inset shows the loading configuration.

The numerical splitting tensile test was conducted using the established mesoscopic model of rock-filled concrete. The boundary conditions and contact properties of the model were defined as follows, as illustrated schematically in Fig 5: Two rigid bearing strips with dimensions of 15 mm × 15 mm × 500 mm were established to simulate the loading strips on the upper and lower surfaces of the specimen; Surface-to-surface contact was defined between the bearing strips and the rock-filled concrete. The tangential behavior followed the penalty friction formulation based on Coulomb’s friction law, while the normal behavior was defined as “hard” contact, with a friction coefficient of 0.6(A detailed parametric analysis was conducted subsequently); A vertical displacement load of 3 mm was applied to the upper bearing strip, while the degrees of freedom in the remaining directions were constrained,the lower bearing strip was fully fixed. The analysis was performed using the Abaqus/Explicit solver, with a total loading time of 0.01s. It is generally considered that the loading process can be regarded as quasi-static when the ratio of kinetic energy to internal energy remains within the range of 5% - 10% [33]. According to the kinetic energy-internal energy time-history curve of the rock-filled concrete model shown in Fig 5, the maximum ratio of kinetic energy to internal energy was 5.53%, confirming that the loading method adopted in this study satisfied the quasi-static loading condition.

**Fig 6:**
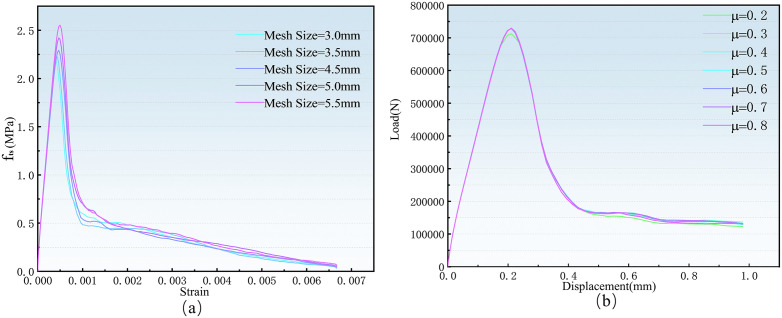
(a) Mesh sensitivity (b) Effect of friction coefficient.

In Abaqus, an appropriate mesh type and mesh size should be selected to improve computational efficiency while ensuring numerical accuracy [34]. Therefore, a mesh sensitivity analysis was required before conducting the numerical simulation tests. Splitting tensile simulations were performed on the cut specimens using mesh sizes of 3 mm, 3.5 mm, 4.5 mm, 5 mm, and 5.5 mm. The corresponding numbers of elements were 3375000, 2146689, 1000000, 729000, and 547709, respectively. The simulated stress–strain curves are shown in Fig 6(a). The post-peak mechanical responses of different specimens were similar, with only minor differences observed in the post-peak stage. The tensile stress increased with increasing mesh size. When the mesh size ranged from 3 mm to 4.5 mm, the relative difference in peak stress was within 5%. Considering both computational accuracy and efficiency, a mesh size of 4.5 mm was selected.

In the RFC splitting tensile simulation, surface-to-surface contact was defined between the bearing strips and the specimen. To determine an appropriate friction coefficient between the contact surfaces, simulations were conducted with different friction coefficients. As shown by the simulation results in Fig 6(b), when the friction coefficient increased from 0.1 to 0.6, the peak load increased accordingly. When the friction coefficient exceeded 0.6, only minor differences were observed among the simulation results, indicating that a friction coefficient of 0.6 was sufficient to represent the relatively strong end restraint [35]. Therefore, a friction coefficient of 0.6 was adopted in the subsequent simulations to reduce the influence of frictional effects on the results.

### 3.2 Material parameter calibration

Lee and Fenves [36] improved the concrete damage plasticity (CDP) model based on continuum damage mechanics. The core assumption of this model is that the failure mechanisms of concrete are tensile cracking and compressive crushing, and it has been widely used to characterize the dynamic and quasi-static mechanical behavior of concrete materials [37–39]. The failure of RFC results from the damage evolution and accumulation of its internal constituent materials, which is similar to that of conventional concrete. Numerous studies have shown that the CDP model can be effectively applied to the analysis of the mechanical behavior of RFC [40,41]. Therefore, the CDP model was adopted to characterize the splitting tensile behavior of RFC. In this constitutive model, isotropic damage variables are used to describe the stiffness degradation process of concrete. The stress-strain relationships under uniaxial tension and compression can be expressed as follows:


$$\begin{array}{c} \begin{array}{c} \sigma_{t}=(1-d_{t})E_{\text{0}}(\varepsilon_{t}-{{\overset{―}{\varepsilon }}_{t}}^{pl})\\ \sigma_{c}=(1-d_{c})E_{\text{0}}(\varepsilon_{c}-{{\overset{―}{\varepsilon }}_{c}}^{pl}) \end{array} \end{array}$$ (1)

where σ_t_ and σ_c_ are the tensile and compressive stresses; ε_t_ and ε_c_ are the tensile and compressive strains; d_t_ and d_c_ are the tensile and compressive damage variables; E_0_ is the initial elastic modulus; and _ε_t_^pl^ and _ε_c_^pl^ are the equivalent plastic strains in tension and compression, respectively.

Experimental studies have shown that the mechanical properties of mortar are similar to those of concrete [42,43], and the ITZ can be regarded macroscopically as a high-porosity cement mortar. Therefore, its mechanical behavior can be characterized by reducing the mechanical parameters of the mortar matrix. This approach is commonly used to simulate the mechanical behavior of concrete materials at the mesoscale, with the reduction ratio generally ranging from 30% to 80% [44–46]. In this study, the tensile strength of the ITZ was calibrated through parametric back-analysis and determined to be 60% of that of the SCC. In addition, although the strength of rock particles is higher than that of mortar, their failure behavior under loading is similar to that of mortar. Thus, the mechanical behavior of rock particles can be characterized by enhancing the mechanical parameters of the mortar matrix [27,35]. Accordingly, the damage plasticity constitutive model was adopted for all mesoscopic constituents of RFC. In the finite element model, the plasticity definition of the CDP model included parameters such as the dilation angle, flow potential eccentricity, f_b0_/f_c0_, K_c,_ and the viscosity coefficient. The specific parameters are listed in Table 3.

**Table 3:** Mesoscopic parameters of constituent materials for RFC finite element model.

Parameter	SCC	ITZ	Rock
Elastic modulus, E (MPa)	23907.104	14344.262	48444.23
Density, ρ(kg/m^3^)	2360.00	2360.00	2730.00
Poisson ratio, υ	0.30	0.30	0.30
Compressive strength, ƒ_c_(MPa)	17.5	10.6	83.77
Tensile strength, ƒ_c_(MPa)	1.7	1.2	8.14
Dilation angle,ψ(º)	30	30	35
Flow potential eccentricity,ε	0.10	0.10	0.10
Stress ratio,σ_b0_/σ_c0_	1.16	1.16	1.16
K_c_	0.667	0.667	0.667

### 3.3 Model validation

**Fig 7:**
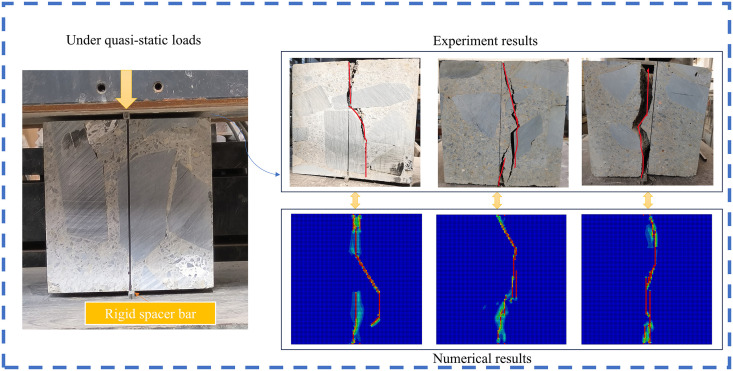
Comparison of the numerical and experimental splitting tensile strengths.

**Fig 8:**
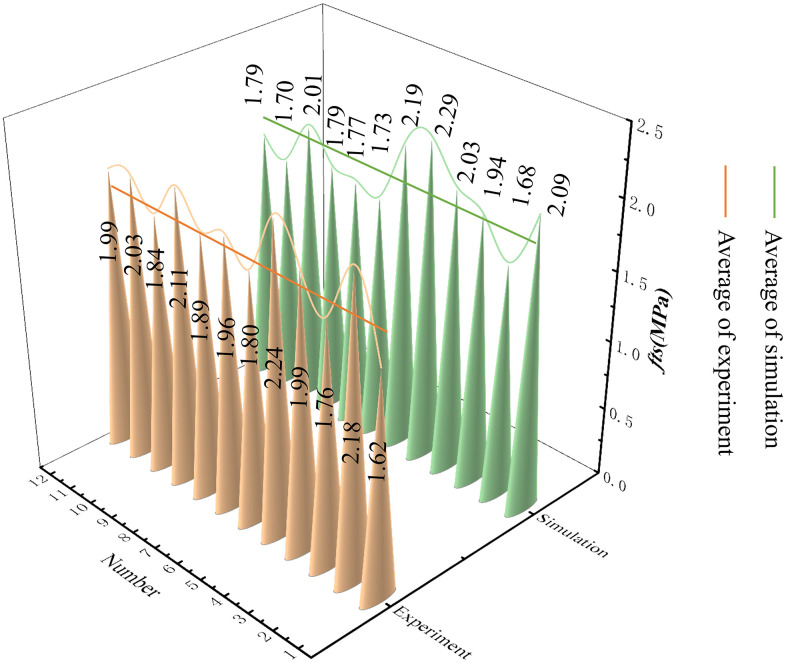
Comparison of the numerical and experimental failure patterns.

Based on the cutting method used in the field test, the RFC specimen with dimensions of 2000 mm × 2000 mm × 2000 mm was cut into cubic specimens with a side length of 450 mm. Twelve specimens were randomly selected and subjected to splitting tensile simulations using the same loading method and boundary conditions as those in the experiment, and the simulation results were compared with the experimental results. Fig 7 presents the comparison of the splitting tensile strength of the 12 specimens. The average simulated tensile strength was 1.92 MPa, with a relative error of 1.53% compared with the experimental result. Meanwhile, the standard deviation of the tensile strength was 0.194 MPa, indicating a dispersion consistent with that observed in the experiment. To further verify the rationality of the mesoscopic parameters, the cracking of the rock-filled concrete model under loading was characterized by elements with tensile damage variable DAMAGET greater than 0.9 [45]. Fig 8 compares the failure characteristics obtained from the simulation and the experiment. The failure morphology of the RFC cubic specimens obtained from the numerical simulation was close to that observed in the laboratory tests, with through-going and irregular main cracks forming along the concentrated loading line. Therefore, the established three-dimensional mesoscopic model can accurately reproduce the splitting tensile process of rock-filled concrete, confirming the rationality of the calibrated mesoscopic parameters.

## 4. Numerical results and analysis

To reveal the failure mechanism of rock-filled concrete under splitting tensile loading and clarify the effects of ITZ strength and rockfill characteristics on its macroscopic mechanical response and damage evolution, parametric analyses were conducted on full-scale specimens using the validated model parameters. This enabled a more accurate characterization of the mechanical behavior of RFC in practical engineering applications.

### 4.1 Failure mechanism

**Fig 9:**
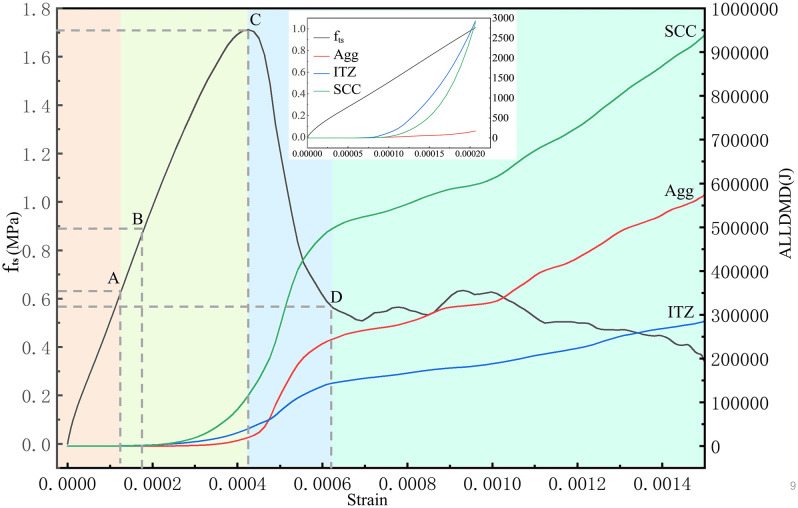
Stress–strain curve and DMD evolution of the three-phase materials under splitting tensile loading.

**Fig 10:**
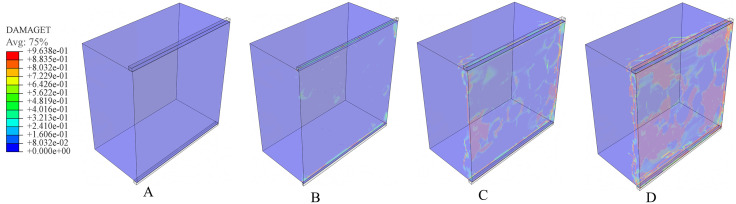
Damage states of RFC specimens at loading points A, B, C, and D.

In Abaqus, damage dissipation energy (DMD) represents the energy consumed during the process of damage evolution under loading. Under applied load, an element is considered to have failed when its damage coefficient reaches 0.9, which allows the simulation of crack initiation and propagation within the mesostructure. Fig 9 presents the stress–strain curves of RFC during splitting tensile loading and the DMD evolution curves of the three phases: Rockfill, ITZ, SCC. Based on the damage dissipation energy and stress evolution of each phase, as well as the damage maps corresponding to characteristic loading points (Fig 10), the splitting tensile process of RFC can be divided into four stages:Linear elastic stage. At the initial loading stage, the DMD values of the three phases remained zero, indicating that no internal damage had occurred in the RFC specimen. The stress curve increased linearly, and the specimen was in a linear elastic deformation state.Yielding stage. As the load increased, the DMD values of the three phases began to rise, indicating the onset of damage initiation. According to the contour maps, within the strain range of 0.64 × 10^−3^ to 2.0 × 10^−3^, the DMD ratio of the ITZ phase started to increase, and its groeth rate was higher than those of the SCC and Rock phase. This indicates that damage first initiated in the ITZ and then propagated rapidly. Subsequently, the DMD of the SCC phase began to increase markedly, suggesting that damage started to extend into the SCC matrix. Meanwhile, the stress curve showed nonlinear growth. Failure stage. After the stress reached its peak, it decreased rapidly. Meanwhile, the DMD values of the ITZ, SCC, and rockfill phases all increased rapidly, indicating that the internal damage of the RFC specimen changed from stable initiation to unstable propagation. Among the three phases, the SCC phase exhibited the largest increase in DMD, followed by the rockfill phase, whereas the increase in the ITZ phase was relatively small. This indicates that cracking of the SCC matrix was the primary cause of main crack formation. During the failure process, damage in the rock phase was mainly associated with local fracture or stress concentration around the rockfill, while the ITZ further promoted crack coalescence.Residual load-bearing stage. The stress entered a fluctuating descending stage, indicating that the cracks had essentially formed and penetrated through the specimen. The load-bearing capacity of the specimen decreased markedly. However, owing to mutual interlocking among the rockfill particles in the rock skeleton, the specimen retained a certain residual load-bearing capacity. Meanwhile, the DMD values of the three phases continued to accumulate, indicating that the cracks continued to propagate and connect. This eventually led to the final failure of the specimen.

### 4.2 Mechanical response

**Fig 11:**
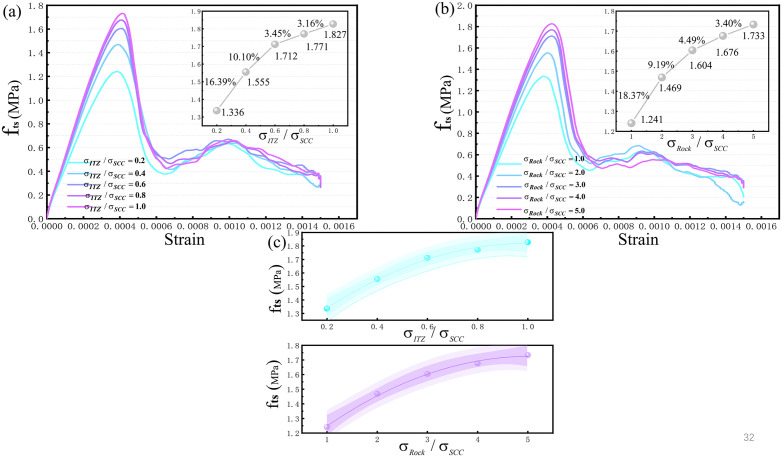
Effects of different parameters on the mechanical response of RFC: (a) ITZ strength; (b) rockfill strength; (c) Variation in splitting tensile strength response.

The effect of ITZ strength on the stress–strain response of the RFC specimens is shown in Fig 11(a). Under different σ_ITZ_/σ_SCC_ conditions, the splitting tensile stress–strain curves of the specimens exhibited similar trends. As σ_ITZ_/σ_SCC_ increased, the slope of the pre-peak ascending branch changed only slightly, indicating that ITZ strength had a limited influence on the initial stiffness of the specimens. In contrast, the slope of the post-peak descending branch increased to some extent, suggesting that a higher ITZ strength accelerated the degradation of post-peak load-bearing capacity and made the brittle failure characteristics more pronounced. As shown in the inset, increasing ITZ strength significantly enhanced the splitting tensile strength of RFC. When σ_ITZ_/σ_SCC_ increased from 0.2 to 1.0, the splitting tensile strength increased from 1.336 MPa to 1.827 MPa, corresponding to an increase of 36.75%. However, when σ_ITZ_/σ_SCC_ increased to 0.8, the increase was markedly reduced to 3.16%, indicating that the strengthening effect induced by further increasing the ITZ strength gradually weakened. This may be because a high-strength ITZ improves the bonding between the SCC and rockfill and enhances the coordinated deformation capacity of the three-phase materials, thereby improving the cracking resistance and load-bearing capacity of RFC. Once the ITZ strength reaches a certain level, it is no longer the dominant weak link controlling the splitting tensile strength of RFC, and the contribution of further ITZ strengthening to the overall strength exhibits a marginal reduction.

The stress–strain response of the RFC specimens under different rockfill strengths is presented in Fig 11(b), and the curve evolution characteristics were similar to those associated with ITZ strength. The strength evolution results further indicate that increasing rockfill strength significantly affected the splitting tensile strength of RFC. When σ_Rock_/σ_SCC_ increased from 1.0 to 5.0, the splitting tensile strength increased from 1.241 MPa to 1.733 MPa, corresponding to an increase of 39.65%. However, when σ_Rock_/σ_SCC_ increased from 4.0 to 5.0, the change in peak stress became markedly smaller, with an increase of 3.40%, indicating a weakened strengthening effect. This suggests that high-strength rockfill can more effectively bear and transfer external loads, enhance the skeleton-supporting effect, and improve resistance to crack propagation, thereby increasing the overall splitting tensile strength of RFC. With further increases in rockfill strength, the crack propagation process was increasingly governed by factors such as the SCC matrix, ITZ bonding performance, and local stress concentration. Consequently, the contribution of rockfill strength to the overall splitting tensile strength exhibited a marginal decreasing trend.Both ITZ strength and rockfill strength exerted a monotonically increasing effect on the splitting tensile strength of RFC, with a threshold behavior observed. As shown in Fig 11(c), both effects can be fitted using Eq. (2) with *R*^2^ greater than 0.996, indicating a good fitting performance.


$$\begin{array}{c} f_{ts}=D+Ce^{-\text{(}\frac{\sigma_{i}}{\sigma_{SCC}}\text{)}/t_{1}}+Be^{-\text{(}\frac{\sigma_{i}}{\sigma_{SCC}}\text{)}/t_{2}}+Ae^{-\text{(}\frac{\sigma_{i}}{\sigma_{SCC}}\text{)}/t_{3}},i:ITZ\text{,}Rock \end{array}$$ (2)

**Fig 12:**
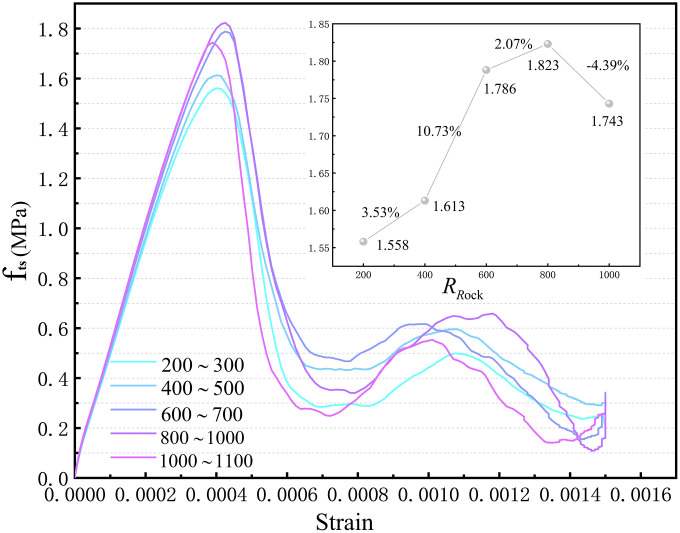
Stress-strain curves of RFC with different rock particle sizes.

**Fig 13:**
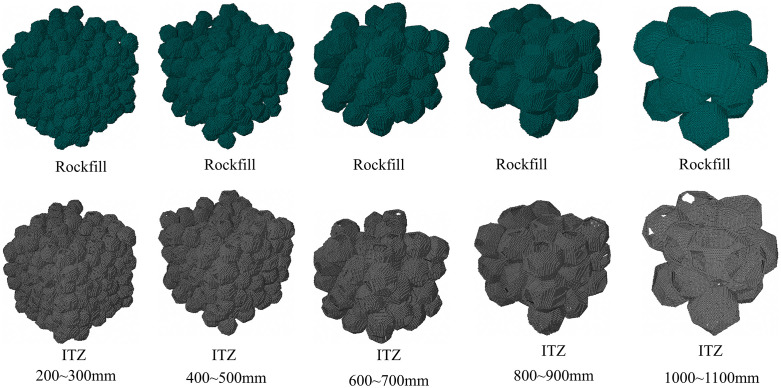
Rock skeleton spatial distributions under different rock particle sizes.

Fig 12 illustrates the influence of rockfill particle size on the splitting tensile stress–strain curves of RFC, and the curve evolution characteristics were similar to those observed for ITZ strength. The results show that the splitting tensile strength of RFC first increased and then decreased with increasing rockfill particle size, rising from 1.558 MPa to 1.823 MPa and then decreasing to 1.743 MPa. The overall increase reached 17.01%, followed by a decrease of 4.39%.Combined with the spatial distributions of rockfill particles with different sizes shown in Fig 13, the number of rockfill particles was larger and their distribution was relatively uniform under smaller particle-size conditions. When the rockfill particle size was moderate, the rock skeleton effect and crack-arresting effect were most pronounced, which could offset the adverse influence of the ITZ interface and improve the splitting tensile strength. When the particle size was excessively large, the number of rockfill particles decreased, and the increased heterogeneity intensified crack localization and local stress concentration, leading to a reduction in strength and more pronounced brittle failure characteristics.

### 4.3 Failure morphology

**Fig 14:**
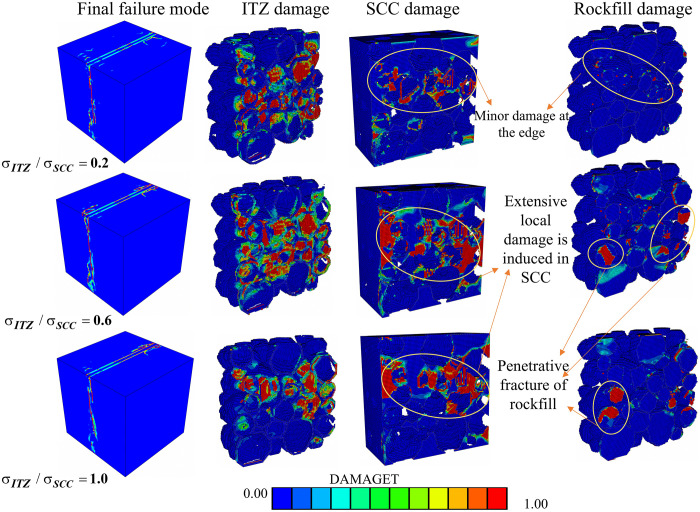
Damage evolution for RFC with different ITZ strengths.

**Fig 15:**
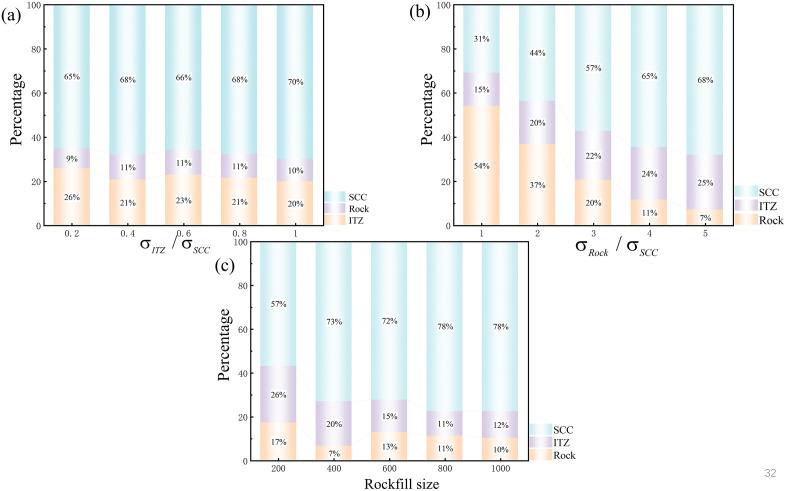
Variations in the relative DMD contributions of Rock, SCC, and ITZ under different parameter conditions: (a) ITZ strength; (b) Rockfill strength; (c) Rockfill size.

The damage nephograms of the three mesoscopic phases and the overall failure morphology of the specimens at σ_ITZ_/σ_SCC_ values of 0.2, 0.6, and 1.0 are presented in Fig 14. As the ITZ strength increased, the specimens ultimately failed through a main crack penetrating along the loading direction, indicating that variations in ITZ strength did not alter the macroscopic failure mode under splitting tensile loading. The damage nephograms of each phase show that ITZ strength had a significant effect on damage initiation and the crack propagation path. When the ITZ strength was relatively low, the ITZ acted as the weak region within the specimen and was more prone to damage. Cracks propagated along the edges of the rock particles and gradually coalesced, while only slight localized damage occurred at the edges of the SCC and rock particles, showing interfacial debonding failure. As the ITZ strength increased, both the degree and extent of ITZ damage decreased, interfacial bonding was enhanced, and cracks rapidly penetrated through the interface. Part of the damage gradually extended into the SCC, while transgranular fracture occurred in the rock particles. Correspondingly, the variation in the DMD proportion of the three phases are shown in Fig 15(a). As ITZ strength increased, the DMD proportion of the ITZ decreased, whereas those of the SCC and rock particles increased. This indicates that, after the interfacial strengthening, damage in the ITZ itself was reduced, and the damage during failure gradually transferred to the SCC and rock particles. Therefore, ITZ strength had a pronounced influence on damage initiation and crack propagation. The dominant failure region of the specimen gradually shifted from the ITZ interface to the joint control of the ITZ and SCC matrix, and the crack propagation path and stress transmission mechanism were consequently altered.

**Fig 16:**
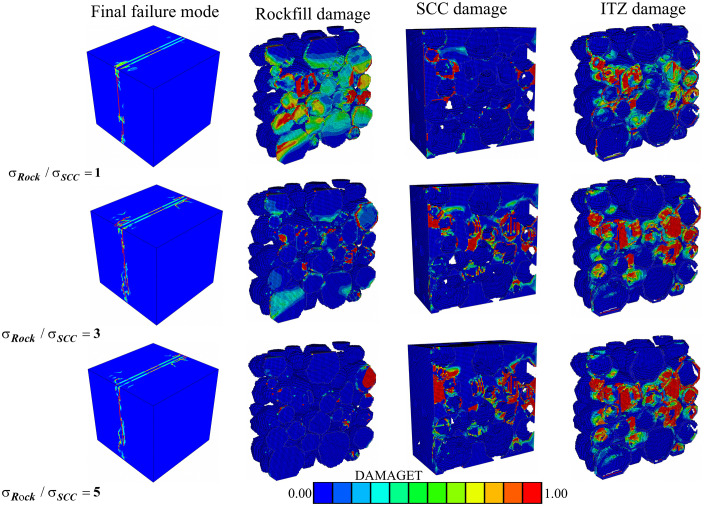
Damage evolution for RFC with different rockfill strength.

Fig 16 illustrates the damage nephograms of the three phases at the peak load, together with the overall failure morphology of the specimens under σ_Rock_/σ_SCC_ values of 1.0, 2.0, and 3.0. At relatively low rockfill strength, substantial damage developed within the rock particles, allowing cracks to penetrate through them, whereas damage in the SCC remained limited. As the rockfill strength increased, the damage in the rock particles decreased markedly. Under the high-strength rockfill condition, the rock particles were damaged only at their edges, and cracks bypassed the rock particles and propagated into the SCC and ITZ. The final failure morphology of RFC was characterized by a through crack along the loading direction. Combined with the variation in the DMD proportion of the three phases shown in Fig 15(b), as the rockfill strength increased, the DMD proportion of the rockfill phase decreased from 54% to 7%, that of the ITZ increased from 15% to 25%, and that of the SCC increased from 31% to 68%. Low-strength rockfill were susceptible to damage under loading, making the specimen more likely to fail through local damage or fracture of the rock particles and thereby limiting the crack-arresting effect of the rock skeleton. In contrast, when high-strength rockfill were used, they were less susceptible to damage, making it difficult for cracks to directly penetrate them. Instead, cracks tended to propagate through the SCC and ITZ.Therefore, increasing the rockfill strength reduced self-damage in the rock particles, enhanced the skeleton effect, and improved the load-bearing and crack-arresting capacities. When the rockfill strength reached approximately four times that of the SCC, the rock particles were no longer the weak link controlling the splitting tensile strength of RFC; the mechanical performance of the specimen was then mainly governed by the SCC and ITZ, exhibiting a threshold effect.

**Fig 17:**
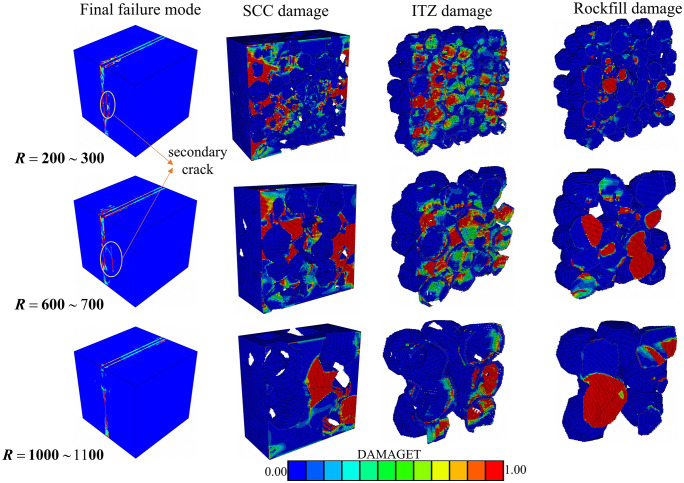
Damage evolution for RFC with different rockfill size.

The damage nephograms of the three phases at the peak load and the overall failure morphology of the specimens with rock-particle sizes of 200 - 300 mm, 600 - 700 mm, and 1000 - 1100 mm are shown in Fig 17. It can be observed that rockfill size had a pronounced influence on the damage propagation path. When the rockfill size was relatively small, the rock particles were uniformly distributed within the specimen, and damage propagated continuously along the loading direction. For large rockfill sizes, the nonuniform distribution of internal rock particles increased the heterogeneity of the specimen, enhanced the crack-arresting effect of the rock particles, and resulted in evident local crack bypassing and damage localization. Meanwhile, variations in particle size affected the final failure morphology of the specimens. Unlike the effects of ITZ strength and rockfill strength, when the particle size was relatively small, a main crack formed along the loading direction and penetrated through the entire specimen, while secondary cracks developed near the centerline of the specimen, leading to a more complex failure pattern. According to the variation in the DMD proportions of the three phases shown in Fig 15(c), the proportion of DMD in the SCC gradually increased with increasing rockfill size, and the post-peak DMD increased more rapidly. This indicates that, as the material heterogeneity increased, the mechanical performance of RFC was mainly governed by the SCC, with more pronounced brittle failure characteristics.

## 5. Relative effects of different factors on the splitting tensile performance of RFC

To comprehensively evaluate the influence degree of different factors on the splitting tensile performance of RFC, ITZ strength and rockfill strength were classified as material strength attributes, whereas rockfill particle size distribution was classified as a mesoscopic geometric attribute. For the material strength attributes, two-way analysis of variance was used to quantitatively compare the significance and sensitivity differences of the effects of ITZ strength and rockfill strength. For the mesoscopic geometric attribute, the effects of single particle size distribution and graded particle size distribution on the splitting tensile performance of RFC were comparatively analyzed by combining stress-strain curves and damage evolution characteristics.

### 5.1 Analysis of material strength attributes

Two-way analysis of variance can be used to quantify the effects of two independent variables on a dependent variable, while also evaluating the effect of their interaction on the dependent variable [47]. Therefore, this statistical method was adopted to analyze the effects of rockfill strength and ITZ strength on the splitting tensile strength of RFC. The rockfill strength ratio was defined as factor A, and the ITZ strength ratio was defined as factor B. Considering that the randomness of the RFC rock skeleton may introduce dispersion into the test results, three different rock skeleton structures were used for repeated simulations under each working condition while keeping the parameters unchanged. The mathematical statistical model is expressed as follows:


$$\begin{array}{c} P_{ijk}=\mu +\alpha_{i}+\beta_{j}+(\alpha \beta {)}_{ij}+\varepsilon_{ijk}\left\{ \begin{array}{c} @c@i=1,2,\ldots a\\ j=1,2\ldots b\\ k=1,2\ldots \text{c} \end{array}\right. \end{array}$$ (3)

Where *P*_*ijk*_ is the *k*-th observation under the combination of the *i*-th level of factor A and the *j*-th level of factor B;μ is the overall mean of all observations when factor effects are not considered; α_*i*_ and β_*j*_ denote the effects of the *i*-th level of factor A and the *j*-th level of factor B, respectively; (αβ)_*ij*_ represents the interaction effect between the two factors; and ε_*ijk*_ is the random error term. It is further assumed that ε_*ijk*_ follows a normal distribution with a mean of 0 and a constant variance.

And the corresponding error decomposition of the model is expressed as follows:


$$\begin{array}{c} SST=SSA+SSB+SSAB+SSE \end{array}$$ (4)

Statistical test hypotheses:


$$\begin{array}{c} H_{0A}:\alpha_{i}=0,H_{1A}:\sum_{i=1}^{a}{\alpha^{\text{2}}}_{i}\neq 0,i\text{=1,2}\ldots a \end{array}$$ (5)

$$\begin{array}{c} H_{0B}:\beta_{j}=0,H_{1B}:\sum_{j=1}^{b}{\beta^{\text{2}}}_{j}\neq 0,j=1,2\ldots b \end{array}$$ (6)

$$\begin{array}{c} H_{0AB}:(\alpha \beta {)}_{ij}=0,H_{1AB}:\sum_{i=1,j=1}^{a,b}(\alpha \beta {{)}^{\text{2}}}_{ij}\neq 0,i\text{=1,2}\ldots a\text{,}j=\text{1,2}\ldots b \end{array}$$ (7)

The degrees of freedom for both factor A and factor B were 3. A significance level of α = 0.05 was adopted, with *F*_0.05_ = 3.86. The analysis results are presented in Table 4.

**Table 4:** ANOVA results of influencing factors.

Mechanical parameter	Difference soure	Mean square	F-value
Tensile strength	Factor A	0.150	33.905
	Factor B	0.318	71.664

According to the ANOVA results, the F-values were 33.905 for factor A and 71.664 for factor B, both reaching the significance level. This indicates that both rockfill strength and ITZ strength had significant effects on the splitting tensile strength of RFC. In addition, *F*_*B*_ > *F*_*A*_, indicating that the splitting tensile strength of RFC is more sensitive to ITZ strength than to rockfill strength. This further suggests that interfacial performance is an important factor controlling the splitting tensile behavior of RFC.

### 5.2 Analysis of mesoscopic geometric attributes

**Fig 18:**
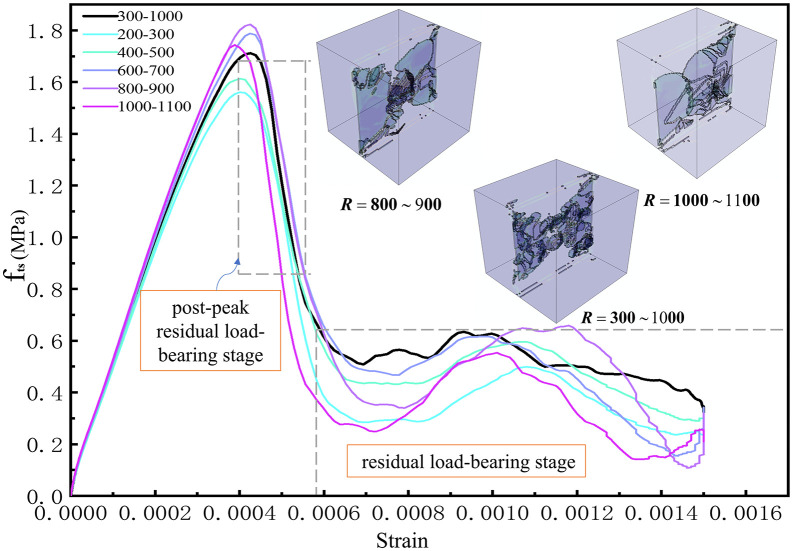
Mechanical performance comparison between single-sized and graded rock particle distributions in RFC.

Fig 18 shows the stress–strain curves of RFC under a single rockfill size and a graded particle-size distribution, with the inset showing the internal damage maps of the specimens at the peak stress. As shown in the figure, the slope of the pre-peak stress-strain curve of the specimen with a graded particle-size distribution was generally consistent with that of the specimens with a single particle size, indicating that the particle-size distribution form had a limited effect on the initial stiffness of RFC. The peak stress of the specimen with a graded particle-size distribution of 300 - 1000 mm was not the highest among all cases. However, compared with the specimens with particle sizes of 800 - 900 mm and 1000 - 1100 mm, its post-peak descending branch was relatively gentle, and it maintained a higher stress level during the residual load-bearing stage.

Combined with the damage maps at the peak stress, the damage in specimens with a single large particle size exhibited pronounced localization, leading to a rapid decrease in post-peak load-bearing capacity. In contrast, in the specimen with a graded particle-size distribution of 300 - 1000 mm, the rockfill jointly participated in load bearing and crack arresting, causing the crack propagation path to become tortuous. The damage region was more dispersed, and the main crack penetration path was more curved. Therefore, a graded particle-size distribution can balance peak strength, post-peak damage development, and residual load-bearing capacity, and its effect on the splitting tensile performance of RFC is superior to that of a single particle size distribution.

The results show that both ITZ strength and rockfill strength significantly affect the splitting tensile strength of RFC, with ITZ strength showing higher sensitivity. Rockfill particle size distribution mainly affects post-peak damage evolution, crack propagation paths, and post-peak bearing stability. Therefore, optimization of the splitting tensile performance of RFC should prioritize improvement of the interfacial performance of the ITZ. On this basis, rockfill strength should be reasonably selected and rockfill particle size distribution should be controlled to enhance crack resistance and improve post-peak failure stability.

## 6. Conclusions

This study conducted splitting tensile tests on RFC specimens cut from a test bin and established an engineering-scale three-dimensional mesoscopic finite element model with a side length of 2000 mm based on the actual working conditions. The effects of ITZ strength, rockfill strength, and rockfill particle size on the splitting tensile performance, failure morphology, and damage evolution mechanism of RFC were analyzed. The main conclusions are as follows:

(1) Under splitting tensile loading, the specimens formed a through-going main crack along the concentrated loading line and failed under the combined effects of SCC matrix fracture, ITZ bond failure, and rockfill through-cracking failure. Owing to the random distribution of rockfill and the nonuniformity of the rockfill-SCC interface, the crack propagation path exhibited pronounced deflection and localization characteristics, resulting in a more complex failure morphology of RFC than that of ordinary concrete.
(2) Both ITZ strength and rockfill strength significantly affected the splitting tensile strength of RFC. Increasing ITZ strength weakened interfacial damage and promoted the transfer of damage from the ITZ to the SCC matrix and rockfill phases. When σ_ITZ_/σ_SCC_ increased from 0.2 to 1.0, the splitting tensile strength of RFC increased by 36.75%. Increasing rockfill strength reduced damage within the rockfill itself and enhanced the load-bearing and crack-arresting effects of the rockfill skeleton. When σ_Rock_/σ_SCC_ increased from 1.0 to 5.0, the splitting tensile strength of RFC increased by 39.65%. In addition, both factors exhibited threshold effects, with corresponding threshold values of approximately σ_ITZ_/σ_SCC_ = 0.8 and σ_Rock_/σ_SCC_ = 4.0. In engineering design, the strength parameters of the ITZ and rockfill should be selected reasonably by considering these threshold values, so as to improve structural performance while accounting for material utilization efficiency and cost effectiveness.
(3) Rockfill particle size distribution had a nonmonotonic effect on the splitting tensile performance of RFC. When the rockfill particle size increased from 200 - 300 mm to 800 - 900 mm, the splitting tensile strength of RFC increased from 1.558 MPa to 1.823 MPa, corresponding to an increase of 17.01%. When the particle size further increased to 1000 - 1100 mm, the splitting tensile strength decreased to 1.743 MPa, corresponding to a reduction of 4.39%. A moderate particle size was beneficial for mobilizing the load-bearing and crack-arresting effects of the rockfill skeleton, whereas excessively large particle sizes enhanced the internal heterogeneity of the material, leading to local stress concentration and localized damage propagation. Compared with a single particle size distribution, a properly graded particle size distribution could optimize the rockfill skeleton structure, promote a more dispersed damage distribution, and improve the post-peak failure stability of RFC.
(4) The comprehensive analysis showed that the splitting tensile strength of RFC was more sensitive to ITZ strength than to rockfill strength, indicating that interfacial quality is a key factor controlling the crack resistance of RFC. In practical engineering, priority should be given to improving the filling compactness of SCC and the interfacial bonding performance, while reasonably selecting rockfill strength and optimizing rockfill particle size gradation, so as to enhance the crack resistance and failure stability of RFC. The results provide a reference for ITZ quality control, rational selection of rockfill strength, and determination of rockfill particle size ranges in RFC material design, and also provide a basis for crack resistance analysis and mesoscopic model parameter selection for engineering-scale RFC structures.

With the increasingly widespread application of RFC in engineering, its tensile performance and failure mechanism require further investigation. Based on an engineering-scale three-dimensional mesoscopic finite element model, this study analyzed the effects of ITZ strength, rockfill strength, and rockfill particle size on the splitting tensile performance and damage evolution mechanism of RFC. Considering the pronounced heterogeneity of the internal structure of RFC, future studies may further focus on factors such as rockfill ratio, rockfill gradation, SCC workability, interface treatment methods, and actual construction conditions, and improve performance evaluation methods from the specimen scale to the actual structural scale.
